# Posterior fossa astroblastoma: a case report of an extremely rare tumor with challenging diagnosis in a child and a review of literature

**DOI:** 10.1007/s00381-025-06768-7

**Published:** 2025-02-14

**Authors:** Ehab Shabo, Saida Zoubaa, Gerrit H. Gielen, Ralf Clauberg, Christian Wispel, Torsten Pietsch, Hartmut Vatter, Sevgi Sarikaya-Seiwert

**Affiliations:** 1 Department of Neurosurgery, Friedrich-Wilhelms-University, University Hospital Bonn, Venusberg-Campus 1, Bonn, 53127 Germany; 2 Department of Neuropathology, DGNN Brain Tumor Reference Center, University of Bonn, Bonn, 53127 Germany; 3 Department of Neuroradiology, University Hospital Bonn, Bonn, 53127 Germany; 4 Section of Pediatric Neurosurgery, Department of Neurosurgery, Rheinische, Friedrich-Wilhelms-University, University Hospital Bonn, Bonn, 53127 Germany

**Keywords:** Astroblastoma, Posterior fossa, Diagnosis

## Abstract

A 7-year-old boy presented to the hospital with recurrent nausea and vomiting over 2 weeks. A cranial MRI revealed a large heterogeneous lesion in the posterior fossa extending from the fourth ventricle to the foramen magnum with contact to the brainstem. The lesion showed moderate diffusion restriction and multiple small cystic components. The child underwent gross total resection. The primary histological findings suggested proliferative active tumor without further definition. The extended histological examination 3 weeks later confirmed the diagnosis of astroblastoma. Due to complete resection and full recovery of the patient, watchful waiting with radiological follow-up was recommended. Astroblastoma is an extremely rare tumor especially in the posterior fossa. However, it should be considered as a differential diagnosis in every tumor presenting the discussed radiological and histological features, especially in young aged patients.

## Introduction

Astroblastoma is a very rare neuroepithelial tumor, typically located supratentorially in the cortex of the cerebral hemispheres. It presents about 0.45–2.8% of cerebral gliomas [1–10]. This tumor usually exhibits a bimodal age distribution, with peak prevalence occurring in children aged 5 to 10 years and in young adults between 21 and 30 years [11, 12]. The rarity of the disease, combined with its imaging and histopathologic similarities to other glial tumors, represents a diagnostic challenge [6]. In this case report, we present an extremely rare case of a 7-year-old male with infratentorial astroblastoma and discuss the clinical course and the challenging diagnosis.

## Case report

A **7**-year-old male presented with recurrent nausea and vomiting over the past 2 weeks, especially at night or early morning. A detailed neurological examination revealed no signs of focal neurological deficit, especially gait ataxia, cranial nerve palsy, or acute papilledema.

**Fig. 1:**
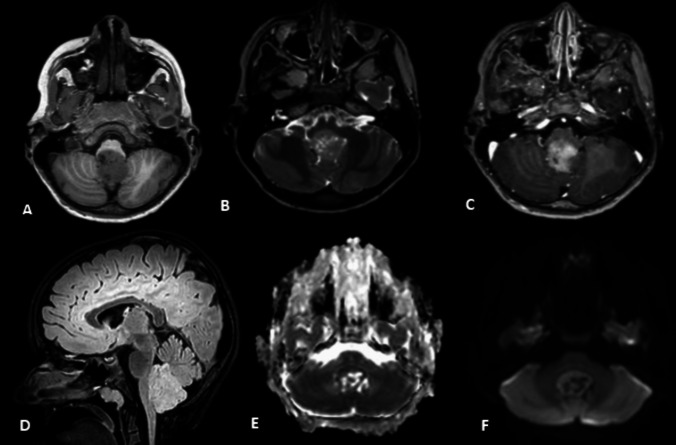
The preoperative MRI findings. **A** T1-weighted axial image shows a heterogeneous hypointense lesion with contact to the brainstem. **B** T2-weighted axial image shows an intermediate heterogeneous lesion with minimal perifocal edema and small cystic components. **C** Axial contrast-MRI with inhomogeneous moderate enhancement of the lesion. **D** Sagittal FLAIR sequence that demonstrates the cranio-caudal extension of the tumor and shows a minimal increase of FLAIR signal intensity. **E**, **F** Apparent diffusion coefficient (ADC) imaging and diffusion-weighted imaging show partly moderate restricted diffusion with multiple small cystic components with proportionately small areas with significant cerebral blood volume increase

A cranial MRI revealed a 2.6 × 2.2 × 3.8 cm heterogeneous, T2-weighted, intermediate, inhomogeneously enhancing lesion in the posterior fossa, extending from the fourth ventricle to the foramen magnum with contact to the brainstem with moderate diffusion restriction and multiple small cystic components. A spinal MRI revealed no further suspicious lesions. Medulloblastoma and high-grade ependymoma were the radiological differential diagnoses (Fig. 1).

An interdisciplinary neuro-oncology board discussed the case. The decision was surgical resection due to the significant mass effect of the tumor and the risk of acute obstructive hydrocephalus.

The boy underwent a successful suboccipital craniotomy and microsurgical tumor resection with intraoperative electrophysiological monitoring and neuronavigation, followed by the placement of a right frontal external ventricular drain (EVD) to manage potential postoperative hydrocephalus. No complications occurred intraoperatively.

Postoperatively, the boy underwent observation at the pediatric intensive care unit overnight. Due to the lack of needed drainage, the EVD was removed on the fourth postoperative day. No postoperative complications or neurological deficits occurred, and the preoperative symptoms were completely resolved.

**Fig. 2:**
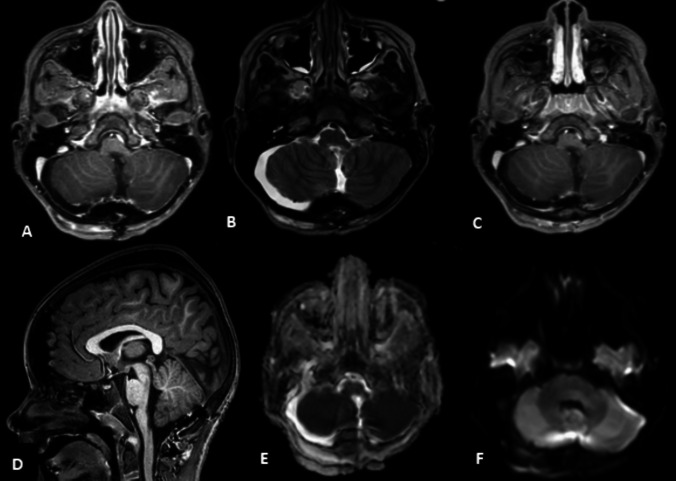
The postoperative MRI findings in the same sequence of Fig. 1 and a total resection with no residual tumor

The radiological postoperative findings showed no evidence of residual tumor or new blood–brain barrier disruption (Fig. 2).

**Fig. 3:**
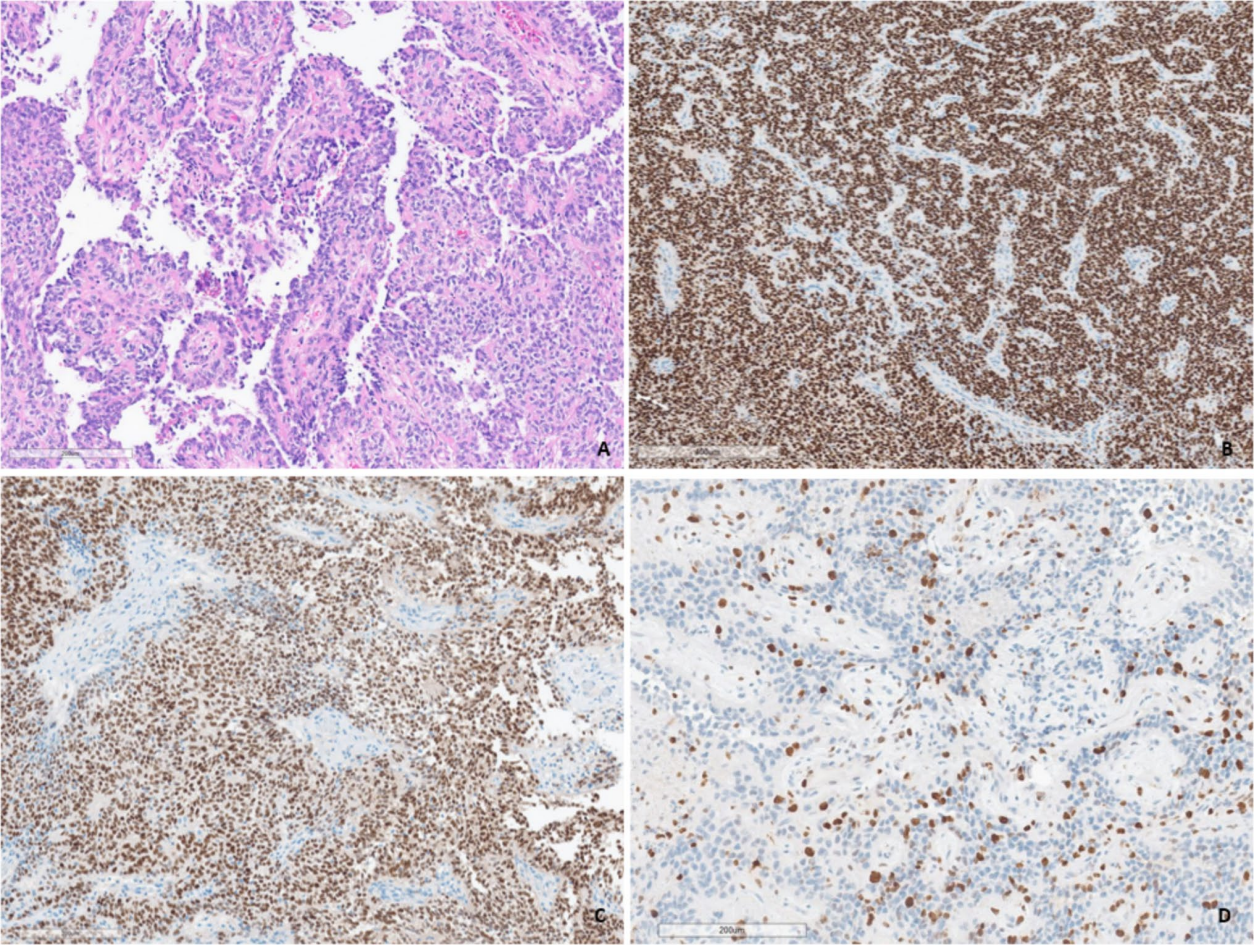
A circumscript glioma composed of round or cuboidal cells arranged around blood vessels in a pseudo-papillary pattern (**A**). Immunohistochemistry displays widespread and strong expression of EMA (**B**). The tumor cell nuclei are immunoreactive for Olig2 (**C**). The Ki67-proliferation index is elevated (**D**)

**Fig. 4:**
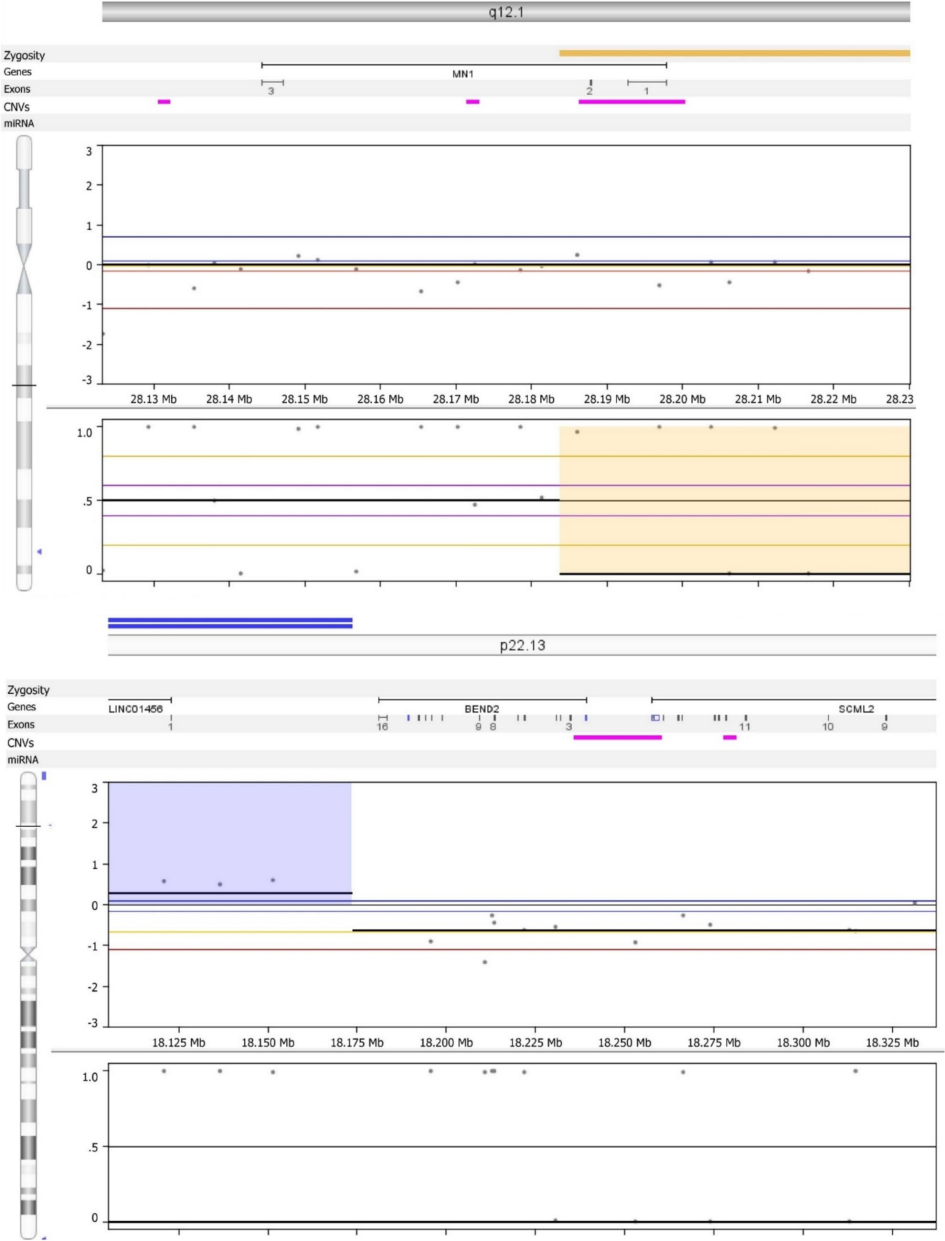
Evidence for a *MN1-BEND2* fusion: Molecular inversion profile technique revealed an in-frame fusion of the MN1 gene at chromosome band 22q12.1 with BEND2 (above) at chromosome band Xp22.13 (below), which is characteristic of astroblastoma, MN1-altered

The primary histological findings suggested a proliferative active tumor with co-expression of epithelial membrane antigen (EMA), oligodendrocyte transcription factor (Olig2), and elevated Ki67-proliferation index (Fig. 3). However, the extended moleculo-histological examination 3 weeks later confirmed the diagnosis of an astroblastoma with MN1-alteration and unmethylated MGMT promoter methylation status (MGMT-STP27) (Fig. 4). In light of the complete resection and full recovery of the patient, the neuro-oncology board recommended a strategy of watchful waiting. This approach included an early follow-up cranial MRI after 4 weeks for reassessment. If no tumor regrowth is detected, the watchful waiting approach would be maintained. However, should any evidence of tumor recurrence occur, further evaluation for surgical intervention and, if necessary, radiation therapy would be considered as the next therapeutic options.

## Discussion

Astroblastoma is a rare tumor of the central nervous system, typically found in the supratentorial region [1–6, 8–10], but in rare cases, it can occur in the posterior fossa, which includes the cerebellum, brainstem, and fourth ventricle [13–18]. Despite the usual peak prevalences of astroblastoma occurring between 5 to 10 and 21 to 30 years in patients [11, 12], it could occur at any age from infancy to 90 s years old [19, 20]. Regarding gender predominance, astroblastoma occurs twice as often in female as in male patients [21].

### Clinical presentation

Patients with posterior fossa astroblastoma often present with nonspecific symptoms related to increased intracranial pressure due to obstructive hydrocephalus or direct brainstem compression [6]. Common symptoms include headaches, nausea, and vomiting (especially in the morning or after lying down), similar to our case. Neurological deficits such as ataxia, cranial nerve palsies, or changes in gait and coordination.

### Radiological features

MRI imaging of astroblastoma typically reveals a well-demarcated, heterogeneous mass often with mixed solid and cystic components. Usually, the tumor is described as hypointense on T1- and hyperintense on T2-weighted images and may enhance with contrast and show moderate diffusion restriction. Calcification is also a common feature in the majority of reported cases [14]. A characteristic imaging feature may be the presence of a “bubbly” appearance, resulting from the solid tumor components intermixed with multiple cysts [8–10]. There is also a mild perifocal edema. Usually, the expansion of edema does not correlate with tumor grade [3–5, 22, 23]. It is worth noting that not all of these findings are consistently observed in every case. Additionally, previous studies have not identified any atypical radiological features beyond those already described in the literature. Additionally, no distinctions in radiological findings have been reported between adult and pediatric populations. In our case illustration, all of these characteristics were present.

### Histological features

Astroblastoma was firstly described by Bailey and Cushing in 1926 and further detailed by Bailey and Bucy in 1930 [24, 25]. In 2016, astroblastoma was categorized with “other gliomas” and was classified into low-grade or high-grade astroblastoma. However, no formal grade for astroblastoma was assigned in the latest “5th” edition of the WHO classification for central nervous system tumors [1].

Astroblastomas are identified by their distinctive perivascular pseudorosettes with nuclear clearing. They show prominent vascularity, eosinophilic granular material, lymphocytic infiltrates, rhabdoid cells, hyaline spherical bodies, and expression of glial and epithelial markers [26, 27].

On immunohistochemical examination, it is typically positive for GFAP, S-100 protein, and vimentin [6, 28]. Methylation profiling is often required for a definitive diagnosis, particularly in complex cases, to distinguish astroblastoma from other high-grade gliomas such as ependymomas or medulloblastomas. MN*-*1 alterations are present in 70% [2]. Table 1 demonstrates a summary of reported cases of posterior fossa astroblastoma.

**Table 1:** Summary of reported cases of posterior fossa astroblastoma

Authors and year	Age(yrs.)/sex	Clinical features	Location	MRI features	Histological features	Treatment	Follow-up/recovery
Brat et al. 2000 [13]	7/M	Circumscribed mass	Midbrain	Circumscribed mass	Pseudorosettes, S-100, GFAP, vimentin, EMA	Total resection, radiotherapy	7 months/full
Ganapathy et al., 2009 [14]	12/F	Headache, vomiting, dizziness, left 5th and 12th nerve paresis, left upper extremity paresis, ataxia and nystagmus	Vermin, brainstem, 4th ventricle	Heavily calcified solid mass with intense enhancement, intracranial parenchymal and spinal leptomeningeal metastases	Ribbon formations, GFAP, EMA, synaptophysin	Subtotal resection, chemotherapy	N/A
Chopra et al., 2007 [15]	37/M	Headache, vomiting, ataxia	Left cerebellar	Heterogeneously enhancing solid-cystic mass	Pseudorosettes, GFAP, S-100, NSE, Ki67 index 5%	Total resection	6 months/full
Shin SA et al., 2018 [16]	11/M	Headache, dizziness, photophobia, gait disturbance	Brainstem	Heterogenous and rim enhancing solid and cystic mass	Rhabdoid tumor features, CK, EMA, vimentin, nestin, p53, S-100, GFAP, Ki67 index 14.4%, MN1-alteration	Subtotal resection, chemotherapy, radiotherapy	N/A
Notarriani et al., 2008 [17]	20/F	Left lower extremity numbness, diplopia, blurred vision, ataxia	Brainstem	Well-circumscribed, contrast-enhancing cystic lesion	Occasional rosettes, EMA, vimentin, GFAP, S-100, Ki67 index 7%	Total resection	3 months/full
Navarro et al., 2005 [29]	3,3/M	Headache, focal findings	4th ventricle	T1-hypointense, T2-hyperintense, solid with calcifications	Pseudorosettes, vimentin, GFAP	Subtotal resection, chemo- and radiotherapy	75 months/full
Yapicier et al., 2019 [6]	4/F	Headache, vomiting, difficulty walking, Papilledema	Left cerebellovermian	Bubbly mass with a mild perifocal edema, T2-iso- and hyperintense, T1- iso- and hypointense with cystic component and calcifications	Pseudorosettes, Ki67 index 3%, GFAP, EMAm S100, Olig2	Total resection, radiotherapy	6 months/full

*CK* cytokeratins, *EMA* epitheliale Membranantigen, *F* female, *GFAP* glial fibrillary acidic protein, *M* male, *N/A* not available, *NSE* neuron-specific enolase, *Olig2* oligodendrocyte transcription factor 2, *yrs* years

The primary histopathological differential diagnoses for posterior fossa astroblastoma include ependymoma, astrocytoma, and angiocentric glioma. Notably, many ependymomas exhibit true ependymal rosettes, a feature typically absent in astroblastomas. Additionally, nuclear immunostaining for Olig2 in tumor cells tends to rule out most cases of ependymoma.

Two reasons can explain the challenging diagnosis of astroblastoma:

1. The absence of pathognomonic clinical and radiological features
2. The need for an extended immuno-/molecule-/histopathological examination, which requires more time even at specialized histological laboratories

In our case, these difficulties lead to a 3-week delay of diagnosis. This delay fortunately did not affect the overall outcome and plan in our case due to the total resection of the tumor and absence of neurological deficits. However, if a gross total resection is not achievable, a delay could affect the overall prognosis and outcome in case of needed adjuvant radiotherapy.

### Prognosis and treatment

The prognosis for patients with posterior fossa astroblastomas can be favorable, especially following complete surgical resection. Radiation therapy, combined with maintenance therapy, could improve outcome, with a survival rate around 80%. Poor prognostic factors are found to be age over 30, male gender, supratentorial location, and present BRAFV600E mutation [20, 27, 30].

The optimal treatment approach is the surgical removal of the tumor, aiming for complete resection followed by adjuvant radiotherapy [21, 31]. Early initiation of radiation therapy in cases of subtotal resection is crucial, as delays may impact overall prognosis. Continued follow-up and adjuvant therapy are essential to managing these patients, given the tumor's aggressive nature.

## Conclusion

Astroblastoma is an extremely rare tumor that may present in the posterior fossa in children. However, it should be considered as a differential diagnosis in every tumor presenting the discussed radiological and histological features, especially in young patients.

## Data Availability

No datasets were generated or analysed during the current study.
